# Supplementary material for: Cerebellar Agenesis and Bilateral Polimicrogyria Associated with Rare Variants of CUB and Sushi Multiple Domains 1 Gene (CSMD1): A Longitudinal Neuropsychological and Neuroradiological Case Study
Source: Int J Environ Res Public Health. 2022 Jan 22;19(3):1224. doi: 10.3390/ijerph19031224 (PMC8835405; doi:10.3390/ijerph19031224)
Supplement: Supplementary file 1 [file ijerph-19-01224-s001.zip › ijerph-1482551-supplementary.pdf]

Supplementary Information

Detailed Description of Neuropsychological Results

Language-Expression

At 5.7 years of age, the lexical expression score was significantly lower than expected for CA normative data (9 correctly named objects) and below the MA normative data. At third evaluation, the lexical expression score was again significantly lower than expected for CA normative data (27 correctly named objects) but slightly below the MA normative data.

In morphosyntactic expression, her score was significantly lower than expected for CA but average for MA. At 11 years of age, however, the performance was significantly lower even for her MA, showing a linguistic performance similar to children between 4 and 5 years of age. Per each evaluation, she showed phonological, articulatory and phonetic difficulties, as well as prosodic deficits.

Language Comprehension

In lexical comprehension, the girl scored significantly lower than expected for CA normative data. Her raw score corresponded with a lexical quotient (LQ) below 65. This score was also slightly below the mean for MA normative data (LQ = 80). At 11 years of age, she showed the same pattern of performance, with a score that was significantly lower than the mean for CA normative data (LQ = below 65) and slightly below the mean for MA (LQ = 80).

In morphosyntactic comprehension, the girl scored on average for CA at 5.7 years of age but scored significantly lower than MA and CA normative data at 11 years of age.

Phonological Awareness

At 5.7 years of age, the girl scored within the range of CA normative data on each task of the Syllabic Blending (between the 10th and 25th percentile) and on the Syllabic Segmentation task (25th percentile). At 11 years of age, however, she scored significantly below both CA and MA (Phonological Blending and Phonological Segmentation task below 5th percentile).

Memory—Short-Term and Working Memory

Verbal

At 5.7 years of age, the girl's verbal short-term memory score was in the average for the CA normative data (25th percentile). However, at 11 years of age, the performance was significantly lower than the mean for the CA normative data (i.e., corresponding to the 1st percentile) and slightly below the mean for MA normative data (i.e., corresponding to the 5th percentile).

Furthermore, at 5.7 years of age, her phonological working memory score was significantly lower than expected for CA normative data (i.e., corresponding to the 2nd percentile). Her performance was compared with those of MA-matched controls, showing no statistically significant difference (t = -1.96, p = 0.08). Such a pattern of performance was confirmed at 11 years of age, with a non-word repetition score in the mean for MA normative data (i.e., corresponding to the 25th percentile).

Visual-Spatial

On the visual short-term memory test, the girl scored below the mean for CA normative data at 5.7 years of age. Her score on this test was compared with those of MA-matched children, but no significant difference emerged (t = -1.101, p = 0.3). At 11 years of age, the girl scored slightly below CA normative data but on average for MA normative

data. Conversely, on the spatial short-term memory test, her score was within the average for CA and MA normative data, at both evaluations.

Memory—Explicit Long-Term Memory

Verbal

Episodic

At the episodic verbal long-term memory test, immediate recall was within the mean for CA normative data (16th percentile) at 5.7 years of age and no significantly differed from the MA-matched group (t = 0.17, p = 0.86). In the delayed recall, her score was significantly lower than expected for CA normative data (raw score was 0, corresponding to the 2nd percentile) and significantly worse than MA-matched controls (t = -2.46, p = 0.04). At 11 years of age, immediate recall was significantly below the mean for CA normative data (below 1st percentile) but on average for MA normative data (corresponding to the 16th percentile). Similarly, in the delayed recall, the performance was lower than expected for CA normative data (raw score 4, corresponding to the 1st percentile) and slightly below the mean for MA normative data (16th percentile).

Semantic

On the semantic memory task, the girl scored below the CA normative data (2nd percentile) at 5.7 years of age. A comparison with MA-matched controls showed that her score was significantly lower than controls' score (t = -2.67, p = 0.02). At 11 years of age, the girl scored below the CA normative data (2nd percentile) and slightly below the MA normative data (5th percentile).

Visual-Spatial

On both the immediate and delayed object learning tests, the girl scored within the norm for CA normative data (in the 25th and 37th percentile, respectively) at 5.7 years of age; however, she scored slightly below the norm for CA normative data (both scores in the 5th percentile) and on average for MA normative data at 11 years of age.

At 5.7 years of age , in the spatial learning test, the girl's performance for the immediate learning was slightly below the mean for CA normative data (9th percentile), but no significant difference emerged (t = -1.23, p = 0.24) in comparison to MA-matched controls. On the delayed recall task, the girl's performance was significantly below the mean for the CA normative data (2nd percentile), but again, no significant difference emerged in comparison to MA-matched controls (**(**t = -1.83, p = 0.09).

Nevertheless, when tested at 11 years of age, she scored in the mean for CA normative data in both immediate and delayed recall (95th and 63th percentile, respectively) and superior to the mean for MA normative data (above 99th and 98th percentile, respectively).

Memory—Implicit Long-Term Memory

Within-group analyses were first conducted for each control group to detect the occurrence of implicit learning after execution of the Serial Reaction Time test (Nissen & Bullemer, 1997; Vicari et al., 2005). Data from the CA-1, CA-2 and MA-2 groups showed an implicit learning effect, whereas data from the MA-1 group did not show reliable measure of implicit sequence learning and thus were excluded from comparisons.

At 5.7 years of age, when the girl's performance was compared with that of CA-1, no difference was found in RTs per block (see Tab.1, part a) (all p >0.05). However, her performance at 11 years of age revealed higher RTs than CA-2 controls per each block. The girl and CA controls were also compared on the implicit learning index (block IV minus block V) by means of the Revised Standardized Difference Test (Crawford & Garthwaite, 2005). Results showed no difference in the girl's performance compared to CA-1 and CA-2 in each evaluation ($t(11)$ = 1.16, p = 0.29; $t(11)$ = 0.78, p = 0.45, respectively). Furthermore, the difference in RTs passing from Block I to block IV (which were found significantly reduced in CA-1 and CA-2) did not significantly differ between the girl's performance at

5.7 years of age and CA-1 ($t(11) = 1.30$, $p = 0.19$); instead, the RTs difference at 11 years of age differed but just because the girl showed a difference more extreme than CA-2 ($t(11) = 5.50$, $p < 0.001$), thus indicating an implicit learning effect.

At 5.7 years of age, the girl's number of errors did not significantly differ from CA-1 (all comparisons $p > 0.10$), but her number of omissions in each block was significantly higher (all $p < 0.01$). Moreover, the difference between her mean number of errors in repeated blocks and random blocks did not differ ($t(11) = 1.51$, $p = 0.16$) from controls (Random blocks: CA-1 = 26 ± 11.8, the girl = 10.5. Repeated blocks: CA-1 = 23 ± 14.8, the girl = 12). Concerning omissions, the girl's performance differed from CA-1's performance ($t(11) = 3.69$, $p < 0.19$), that is, her performance showed a greater difference between random and repeated blocks than the controls' performance (Random blocks: CA-1 = 3 ± 2, the girl = 15. Repeated blocks: CA-1 = 1 ± 1.9, the girl = 9.7). However, at 11 years of age, both the girl's number of errors and omission did not significantly differ from CA-2 (all comparisons $p > 0.10$). Again, the difference between repeated blocks and random blocks for the mean number of errors ($t(11) = 1.51$, $p = 0.16$) and omissions ($t(11) = 1.51$, $p = 0.16$) did not differ between the girl and CA-2 (Errors: Random blocks CA-2 = 6 ± 5.7, the girl = 1; Repeated blocks CA-2 = 5 ± 3.9, the girl = 3. Omissions: Random blocks CA-2 = 0.7 ± 0.9, the girl = 1; Repeated blocks CA-2 = 0.7 ± 0.8, the girl = 1).

Data were analyzed by means of repeated measures ANOVAs with block (from I to V) as within factor. In both the CA-1- and CA-2-matched group, results of median reaction times (RTs) revealed a significant block effect (CA-1: $F(4,44) = 12.8$; $p < 0.001$, CA-2: $F(4,44) = 3.92$; $p < 0.01$), demonstrating a different pattern of RT changes across blocks. Post hoc analysis (Tukey HSD test), carried out to qualify this effect, revealed an RT decrement passing from block I (CA-1: 829 ± 171 msec; CA-2: 471 ± 61 msec) to block IV (CA-1: 528 ± 156 msec, $p < 0.001$; CA-2: 405 ± 77 msec, $p < 0.001$). Critically, RTs increased significantly passing from block IV to block V (CA-1: from 405 ± 77 msec, to 727 ± 161 msec, $p < 0.01$; CA-2: from 405 ± 77 msec, to 442 ± 55 msec, $p < 0.05$). This difference is usually considered the most reliable measure of implicit sequence learning. Conversely, results of MA-1-matched group revealed no learning effect because RTs on the five blocks did not differ ($F(4,36) = 1.77$; $p = 0.15$). Given that MA-1-matched controls showed no learning effect (as revealed by the RT measure), this group was excluded from implicit learning task comparisons. However, MA-2 showed implicit learning effect. Indeed, a significant block effect emerged in the median RTs (MA-2: $F(4,44) = 15.27$; $p < 0.001$), with RTs decrement passing from block I (MA-2: 758 ± 174 msec) to block IV (MA-2: 598 ± 169 msec, $p < 0.001$) and RTs increment passing from block IV to block V (MA-2: from 598 ± 169 msec to 743 ± 164 msec, $p < 0.01$).

We also analyzed the number of errors and omissions in the control groups. We conducted separate repeated measure ANOVAs with Type of blocks (mean number in random blocks, i.e., I and V, and in repeated blocks, i.e., II to IV) as within factor. Results in CA-1 showed a Type of blocks effect, which bordered on statistical significance, on errors ($F(1, 11) = 4.03$; $p = 0.07$) and a significant Type of blocks effect on omissions ($F(1,11) = 8.13$; $p = 0.02$); in particular, more errors and omissions were made on stimuli belonging to the random (I,V) than the repeated blocks (II-IV) (Errors: Random blocks CA-1 = 26 ± 11.8; Repeated blocks CA-1 = 23 ± 14.8. Omissions: Random blocks CA-1 = 12 ± 1.8; Repeated blocks CA-1 = 2 ± 1.8). However, no such effects emerged in the CA-2 group, probably due to a ceiling effect in both measures (Errors: Random blocks CA-2 = 6 ± 5.7; Repeated blocks CA-2 = 5 ± 3.9. Omissions: Random blocks CA-2 = 0.7 ± 0.9; Repeated blocks CA-2 = 0.7 ± 0.8). In the MA-2, Type of blocks effect also emerged on errors ($F(1, 11) = 15.15$; $p < 0.01$) and omissions ($F(1, 11) = 7.99$; $p < 0.05$) with higher errors and omissions in the random than the repeated blocks (Errors: Random blocks MA-2 = 17.5 ± 10.1; Repeated blocks MA-2 = 10.6 ± 8.1. Omissions: Random blocks MA-2 = 5.6 ± 5.2; Repeated blocks MA-2 = 1.6 ± 2.1).

Executive Functions

Attention

The girl's score on a visual the selective attention task was below the mean for CA normative data (3rd percentile) but within the average for MA normative data. Moreover, on the sustained visual attention test, she performed below the mean for CA normative data (2nd percentile) and slightly below the mean for MA normative data (between the 5th and 10th percentile). At 11 years of age, on the selective visual attention test, the performance was significantly below the MA normative data (below the 1st percentile), whereas the performance on sustained attention task was on average for MA normative data (16th percentile).

Planning

The girl scored far below the mean for CA normative data (below the 1st percentile) and below the mean for MA normative data (below the 2nd percentile) at 5.7 years of age as well as at 11 years of age (both below the 5th percentile).

Inhibition

On the Go/No-Go task, the girl's meRTs in the Go condition and in the No-Go condition did not differ from those of CA-1-matched controls (Go condition, $t = 1.47$, $p = 0.16$; No-Go condition, $t = 0.51$, $p = 0.62$) and MA-1-matched controls (Go condition, $t = 0.012$, $p > 0.10$; No-Go condition, $t = -0.263$, $p > 0.10$). Her errors in the No-Go condition also did not significantly differ from those of CA-1-matched ($t = -0.23$, $p = 0.82$) and MA-1-matched ($t = -0.411$, $p > 0.10$) controls. However, her number of omissions exceeded the tolerance limit in both CA-1-matched ($t = 13.36$, $p < 0.001$) and MA-1-matched ($t = 12.447$, $p < 0.001$) comparisons.

At 11 years of age, the girl's meRTs in the Go condition and in the No-Go condition were significantly different from CA-2-matched controls (Go condition, $t = 3.402$, $p = 0.005$; No-Go condition, $t = 2.33$, $p = 0.03$), but they did not differ from those of MA-2-matched controls (Go condition, $t = 1.113$, $p > 0.28$; No-Go condition, $t = 0.15$, $p > 0.88$). The girl's errors in No-Go conditions significantly differ from those of CA-2-matched ($t = 7.68$, $p < 0.001$) and MA-2-matched ($t = 2.63$, $p > 0.01$) controls.

However, her number of omissions did not differ from both CA-2-matched ($t = 0.524$, $p = 0.61$) and MA-2-matched ($t = -0.826$, $p = 0.42$) comparisons.

Perceptual and Visual-Spatial Abilities

Visual-Motor Integration

The girl reached a quotient of 69, which was below the expectations for CA normative data, and an equivalent age of 3.6 years in line with her MA. At 11 years of age, she reached a quotient of 62, in line with her MA.

However, on the Perceptual subtest, the girl reached a quotient of 85, which was within the average for CA normative data, and an equivalent age of 4.5 years. At 11 years of age, the girl reached a quotient of 65, which was below the expectations for CA normative data, and an equivalent age of 5.2 years

At 11 years of age, the girl reached a quotient of 62, which was below the expectations for CA normative data, and an equivalent age of 5.6 years, in line with her MA. Finally, on the Motor Coordination subtest, she reached a quotient of 75, with an equivalent age of 4.6 years (slightly below the mean for CA normative data but on average for MA normative data). At 11 years of age, she reached a quotient of 65, which was below the expectations for CA normative data, with an equivalent age of 5.2 years (on average for MA normative data).

Perceptual Abilities

On the subtest concerning the perception of figures in different spatial positions, the girl scored within the average range for CA normative data (25th percentile) at 5.7 years of age but below the mean for CA normative data (2nd percentile) and on the mean for MA normative data (25th percentile) at 11 years of age. On the subtest that involved discriminating the figure on a confounding background, her performance was also in line with the

mean for CA normative data at 5.7 years of age (16th percentile) and 11 years of age (75th percentile).

Academic Abilities

Severe deficit in both reading and math abilities were detected, although only tasks tailored for her MA were considered for the assessment.

Reading and Writing Abilities

At 8 and 11 years of age, the girl was impaired in reading short sentences and was only able to read bi-syllabic words. Not-structured evaluation was possible. At 8 years old, she was able to write her own name and high frequency bi-syllabic words and manifested difficulty with handwriting. At 11 years of age, the girl exhibited marked difficulty at dictation task, even below that expected for her MA (2/16 corrected responses).

Math Abilities

At 8 years of age, counting abilities were on average for the girl's MA, considering norms for the first intermediate grade (errors Z score =+0.2; Time Z score +0.4). Moreover, number dictation was on average (errors Z score =-0.3), whereas quantity discrimination ability was less than average (Z score =-2.2). At 11 years old, her performance on counting was in line for MA, considering normative data for the first grade (errors Z score =-0.2; Time Z score +0.3), as well as the performance in number dictation (errors Z score 0); however, on mental calculation and quantity discrimination, she showed marked impairment and was not able to complete the tasks.

Adaptive Behavior

At first evaluation, the girl's IQ-equivalent scores were within the average or above those expected for MA and IQ (respectively: Communication = 113, daily living skills = 112, socialization = 129 and motor skills = 116). Consistently, the scores at 8 years old were on average for MA and IQ (respectively: Communication = 114; Daily living skills = 90; Socialization = 107; Motor abilities = 107). When the girl was 11 years old, scores at each subscale were again on average for her MA and IQ (respectively: Communication = 103; Daily living skills = 101; Socialization = 107; Motor abilities = 106).

Differently from the previous evaluations, at 15 years of age, her scores at each subscale were significantly below that expected for CA (Global IQ-equivalent score = 20) and for MA (Global IQ-equivalent score = 76). The girl's scores on the four domains for MA are reported: Communication = 84; Daily living skills = 78; Socialization = 68; Motor abilities = 90.

Compared to the previous assessments, a decline emerged in a number of daily life skills, such as teeth brushing and the use of buttonholes and buckles to get dressed.

Psychopathological Symptoms

No autistic-like symptoms nor significant psychopathological signs emerged at clinical evaluation when the girl was 5.7 years old as well as no clinical results at the K-SADS interview. When the girl was 8 years old, traits of generalized anxiety and oppositional behaviors emerged, supported by K-SADS interview. No clinical psychopathological results were detected by CBCL (see below). When she was 11 years old, psychopathological evaluation revealed the presence of phonophobia-related behaviors, associated with psychological distress and avoidance behavior, traits of generalized anxiety and attention deficit and hyperactivity disorder. At 15 years of age, it was diagnosed a Disruptive Mood Dysregulation Disorder, associated with frequent episodes of extreme behavioral dyscontrol, often evolving into outwardly directed aggressiveness. The girl was much less collaborative, with strongly reduced attention times and global difficulties in starting new activities.

Clinical results similarly emerged at the K-SADS interview and the CBCL questionnaire.

Detailed results of the CBCL questionnaire, administered to the mother, are as follows. No clinical scores were detected at 5 years of age, whereas at 8 years of age a borderline score was reported in the subscale "social competences" (T score = 33), together with a clinical score in the "school" subscale (T score = 27). Over time the psychopathological characteristics worsened, with the increase in clinically relevant or borderline scores. At 11 years of age, borderline scores were registered in the "Activity", "Attention" and "Total Problems" subscales (T scores: 31; 66; 63); clinical scores were reported for the "Social competences", "School", "Total Competence", "Social Problems", "Anxiety" and "Post-traumatic Stress Disorder" subscales (T scores: 21; 24; 19; 72; 73; 72).

At 15.5 years of age, in withdrawn/depressed subscale, internalizing and externalizing problems, Attention Deficit/ Hyperactivity Problems and conduct problems, T-scores increased up to 1 standard deviation from the previous evaluation. For aggressive behavior, oppositional-defiant problems and affective problems, T-scores increased up to 1.5 standard deviation (at 11 years old and 15 years old).

Such behavioral worsening reported by parents was also detectable during the assessment. The girl was remarkably less cooperative during the evaluation, exhibiting higher frustrability, marked reduction in attention times, higher difficulties in starting new tasks and the general tendency in having behavioral dyscontrol during the examination.

Table S1. *CSMD1 (NM_033225.5)* SNPs or rare variants associated with neuropsychiatric and/or cognitive phenotypes.

***CSMD1* rare variants/SNPs**	**NEUROPSYCHIATRIC AND COGNITIVE PHENOTYPE(S)**	**REFERENCES**
p.Arg1962His<br>p.Gly2987Arg	Idiopathic Parkinson	Ruiz-Martínez et al. 2017
rs12681349<br>rs10503253<br>rs1983474	Idiopathic Parkinson	Bai et al 2021
Pro2262Ala<br>Gly827Asp	Autism<br>Schizophrenia	Cukier et al. 2014
rs10503253	Schizophrenia<br>Cognitive abilities<br>Executive functions<br>Monoaminergic transmission	Donohoe et al. 2013, Rose et al 2013, Koiliari et al. 2014, Luykx et al. 2014
rs664600<br>rs4876061<br>rs7017888<br>rs7011965<br>rs10094093<br>rs24886<br>rs13249525	Schizophrenia	Håvik et al. 2011
rs2623659	Schizophrenia	Sakamoto et al. 2016
rs2740931	Episodic memory	Athanasiu et al. 2017
rs2616984	Cognitive function	Stepanov et al. 2017
rs1154037<br>rs4875310	Bipolar disorder	Xu et al 2014, Sklar et al 2008
rs779105<br>rs57812884	Bipolar disorder	Baum et al 2008
rs71534387	Drug addiction phenotype	Drgonova et al. 2015
p.Thr799Ser	Anorexia Nervosa	Bienvenu et al 2019
